# The Excellent Mechanical Performance of Polymer-Coated Ceramsite Particles for Efficient Fracturing: An Explanation from a Surface, Tribological Perspective

**DOI:** 10.3390/ma17010241

**Published:** 2024-01-02

**Authors:** Mengqi He, Jiangkuan Wang, Bin Wang, Yuxi Wu, Ling Wang, Yanbao Guo

**Affiliations:** 1 Liaohe Oilfield (Panjin) Gas Storage Co., Ltd., Panjin 124000, China; hemengqi0@163.com (M.H.); wangjk@petrochina.com.cn (J.W.); wangling071@petrochina.com.cn (L.W.); 2 College of Mechanical and Transportation Engineering, China University of Petroleum, Beijing 102249, China; cupwangbin@163.com (B.W.); 2023210704@student.cup.edu.cn (Y.W.)

**Keywords:** hydraulic fracturing, polymer-coated ceramsite particles, friction and wear properties, better hydrophobicity, high-pressure resistance

## Abstract

Hydraulic fracturing using micro-particles is an effective technology in the petroleum industry since the particles facilitate crack propagation of the shale layer, creating pathways for oil and gas. A new kind of polymer-coated ceramsite particles (PCP) was generated. The friction and wear properties of the particles under different loads and speeds were also studied. The tribological relationship between the newly fabricated polymer-coated ceramsite particles and the fracturing fluid was studied through tribological experiments under the condition of fracturing fluid lubrication. The results show that, in contrast, the wear of the new-generation particles is relatively stable, indicating that it has good adjustable friction properties. In addition, under the lubrication condition of fracturing fluid, the new-generation particles have better hydrophobicity, high-pressure resistance, and low reflux rate, which have an important value as a practical engineering application for improving shale gas production efficiency and production.

## 1. Introduction

Hydraulic fracturing is an effective technique to improve oil recovery in the petroleum industry [1,2,3,4], which is the process of pumping a fracturing fluid (typically composed of water, particles, and certain chemicals) into a wellbore at very high pressures to rupture rocks and create many fractures [5,6,7,8]. While the fracture crack is formed, many particles (called proppants) are pumped into the fracture along with the fracturing fluid at a high flow of about 15~16 KL/min [9] and rapidly migrate to the crack and become stuck in the crack. Thus, particles trapped in cracks create space, which accelerates crack propagation and increases shale oil production [10,11,12].

In recent years, many researchers have put a lot of effort into researching proppant particles. One of the most widely used forms of polymers in the oil and gas industry is related to proppant coatings in hydraulic fracturing (HF) operations. Proppants are small spheres that must be strong enough to withstand high closing stresses. The application of polymers in proppant coatings has only just emerged in recent years, and by considering strength and flexibility, it is expected to obtain higher performance. Coating the proppant with a thin layer of polymer will result in higher conductivity at the break, greatly reducing the generation of fine powder and fouling, thereby improving the quality of HF treatment. For transporting distance, backflow resistance, and gas/oil conductivity [13,14], Hussain et al. developed a coating particle made of a granular substrate coated with resin and fiber materials to prevent sand or other particles from backflowing [15]. Burukhin et al. developed a new type of interconnected bonding structure to enhance particle backflow control [16]. Monastiriotis et al. improved performance by adding a polymer coating to the particle’s surface [17]. McCrary et al. studied a polyurea-type coating particle with high-temperature and pressure resistance [18,19]. The above studies focused on improving the physical and chemical properties of particles, so the explanation from the mechanical and tribological point of view was insufficient.

In previous research, we have studied the contact behavior between sand particles and sand cleaning tools in the oil/gas industry from the perspective of tribology [4,20,21]. Due to the increasing demand and production of shale oil and gas, the characterization of proppant particles is essential to prove and demonstrate that the shale bed cracks are closely related to hydraulic fracturing [22]. This paper developed polymer-coated particles (PCP) with excellent mechanical properties considering the fracture process and fracture environment.

Therefore, the above-mentioned mechanical properties of PCP were studied and analyzed from the viewpoint of tribological principles.

## 2. Material Methods and Characterization

### 2.1. Experimental Materials

Before the experiment, each shale, ceramsite particle, and polymer-coated particle sample are required. Ceramsite particle was purchased from Xinmi Wanli Industrial Development Co., Ltd., Zhengzhou, China, Bosco Polymers (Shanghai) Co., Ltd., Shanghai, China, provided Polymer coating, and China National Petroleum Corporation provided shale layer. Optical microscope equipment was supplied by Keens Ltd. The shale sample was cuboids with dimensions of 50 mm × 30 mm × 5 mm, and the surface was sequentially polished with 800-, 1500-, and 2000-grit sandpapers. The Young’s Modulus (YM) and Poisson’s Ratio (PR) of shale are 32.4 GPa and 0.23, respectively [19]. The particles are applied with common ceramsite particles (CP) and polymer-coated ceramsite particles (PCP), of which the polymer coating consists of C_8_H_8_O_2_, (C_11_H_12_O_3_)_n_, and C_5_H_6_O_2_ as a ratio of 3:1:1. The size of CP and PCP is about 380~460 μm. The YM and PR of CP are 5.3 GPa and 0.16. At the same time, the YM and PR of PCP are 3.4 GPa and 0.34. The roughness of shale rock, CP, and PCP are 5.23 ± 2.74 μm, 2.67 ± 0.32 μm, and 1.93 ± 0.17 μm. Before the test, the experimental samples were washed with petroleum ether to remove the surface impurities, then soaked in alcohol for 20 min and cleaned repeatedly with deionized water (DI water). Finally, the samples were placed in an oven at about 50 °C for drying. To effectively simulate the fracturing process, a 0.6 wt% guar gum solution (GG solution) is used as the fracturing fluid.

### 2.2. Tribological Apparatus

**Figure 1:**
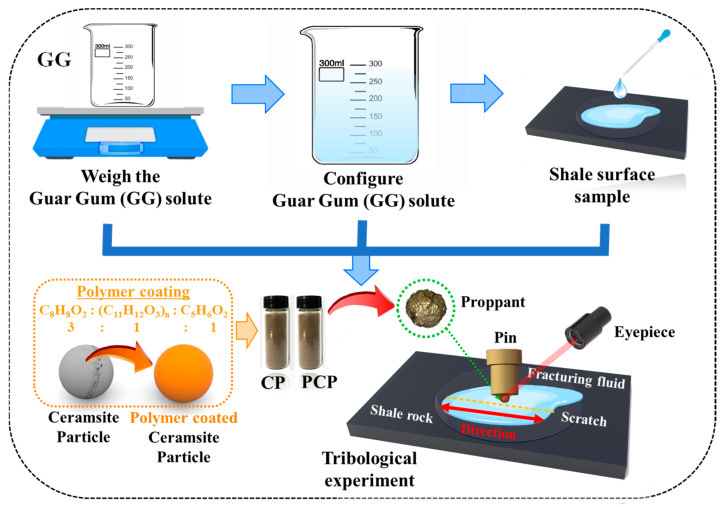
The schematic diagram of tribological experimental procedure between the particle (CP and PCP) and shale rock.

To conduct this study, the ball-on-disc reciprocating tribometer was selected for tribological experiments [23]. As shown in Figure 1, after fixing a single particle to a pin, the pin is stuck to a vertical beam. By using the same principle, a shale sample is stuck to the test platform. The data collection frequency is set at 30 Hz. This experiment is carried out at an average temperature of 25 °C, using a form of dry friction and wet friction (0.6 wt% GG as friction medium) to study the properties of polymer-coated proppants [24].

In practical applications, load and sliding speed are both key parameters that affect the ability of particles to enter cracks quickly and to effectively support cracks by entrapment between crack spaces. Therefore, in order to elucidate the relationship between key parameters and shale crack, five normal loads (0.5 N, 1 N, 1.5 N, 2 N, and 2.5 N) and four sliding speeds (20 mm/s, 35 mm/s, 50 mm/s, and 65 mm/s) were used in tribological experiments. Each group of experiments with the same parameters was repeated three times, and the data were recorded. The surfaces of samples were measured by 3D profilometer (TDP) and SEM.

### 2.3. Material Characterization

**Figure 2:**
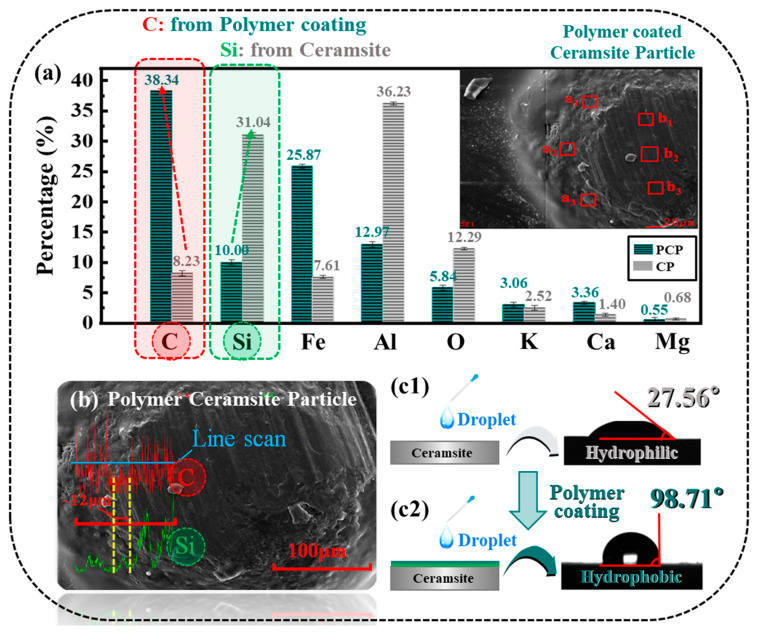
Characterization of PCP and CP: (**a**) Element percentage of PCP and CP materials (areas a1, a2, and a3 are the regions of PCP, and areas b1, b2, and b3 are the regions of CP). (**b**) SEM images of transient change curves of C and Si elements in the critical region. (**c1**) Contact angle diagram of CP surface. (**c2**) Contact angle diagram of PCP surface.

In this paper, PCP and CP were compared in chemical element ratio and schematically shown in Figure 2a, and it was confirmed that PCP showed a higher amount of carbon due to the coating [25]. PCP with some peeling of the coating was selected, showing the difference between the substrate layer and the coating layer. Three observation ranges, a1b1, a2b2, and a3b3 (a1, a2, and a3 are the parts of polymer coating film, and b1, b2, and b3 are the parts of substrate layer), were selected to measure the percentage of chemical elements, then the obtained data are averaged to confirm for the presence of polymer coating. Preferentially, the results show that the carbon (C) element dominates in the coating, and silicon (Si) is the dominant element in the CP (substrate). The variation in the percentage of chemical elements (C and Si) crossing a line area is shown in Figure 2b. The length of the abrupt change in the C-Si element percentage curve at the critical position between the coated and uncoated regions was about 12 μm.

Hydrophilicity and hydrophobicity can be indirectly demonstrated by measuring the contact angle between the surface of the material and a droplet of the fracturing fluid. As shown in Figure 2(c1,c2), the contact angle between the polymer-coated material and the water droplets is 98.71°, and the contact angle between the CP material and the water droplets is 27.56°, indicating that it can be seen that when the polymer coating is applied, the material properties change from hydrophilic to hydrophobic [26,27,28].

### 2.4. Polymer Coating Thickness Analysis

**Figure 3:**
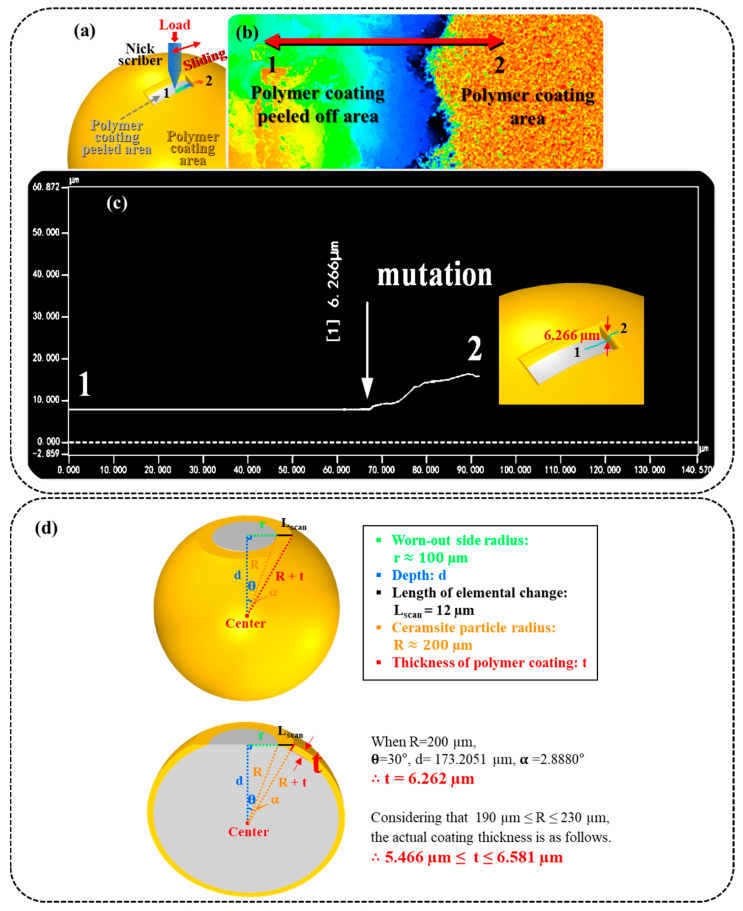
The calculation principle of polymer coating film thickness: (**a**) Schematic diagram of the scratch test. (**b**) Linear region surface. (**c**) The height difference curve between the two zones. (**d**) Diagrams and formulas of PCP cross-sections for calculating coating thickness.

In addition, to measure the thickness of the polymer coating, a portion of the polymer film was removed using a scratch meter until the underlying layer was visible. Afterward, the depth of the scratch was observed by a three-dimensional profilometer (Figure 3a). The line area of the surface 1–2 (1 is the non-coating area, 2 is the coating film area) is shown in Figure 3b. By comparing the elevations of the two regions, a change in the height curve can be observed, and these data show that the height difference between the two regions is about 6.266 μm (Figure 3c). Through this developed method, the thickness of the coating layer can be obtained simply. To verify the accuracy of the developed method, the worn PCP surface (100 μm area) was scanned by an electron microscope line, and the range of chemical elemental change was measured to be 12 μm (L_scan_) in Figure 3d. Also, the worn-out side radius (r) was measured to be about 100 μm, and the ceramsite particle radius (R) was measured to be about 200 μm. The angle between ceramsite particle radius and worn-out side radius is calculated as follows:

(1) $$\theta ={sin}^{-1}(\frac{r}{R})={sin}^{-1}\left(\frac{100}{200}\right)=30{}^{\circ},$$

Also, the depth is calculated as follows:

d = R cos θ = 200 cos 30° ≈ 173.2051,(2)

The length of polymer coating thickness is approximately 6.262 μm calculated by the following formulas.

(3) $$\theta +\alpha ={tan}^{-1}(\frac{r+Lscan}{R})={tan}^{-1}\left(\frac{100+12}{173.2051}\right)\approx 32.8880{}^{\circ},$$

(4) $$cos(\theta +\alpha )=\frac{d}{R+t}$$

(5) $$t=\frac{d}{cos(\theta +\alpha )}-R=\frac{173.2051}{cos(32.8880{}^{\circ})}-200\approx 6.262$$

Similarly, when the size distribution of PCP is 380~460 μm, the polymer coating thickness shows a variation of 5.466~6.581 μm. By confirming that the 6.266 μm coating thickness obtained by the scratch tester is within the calculated range, it was confirmed that the scratch tester measurement was accurate.

## 3. Results and Discussion

### 3.1. Tribological Behaviour of PCP

**Figure 4:**
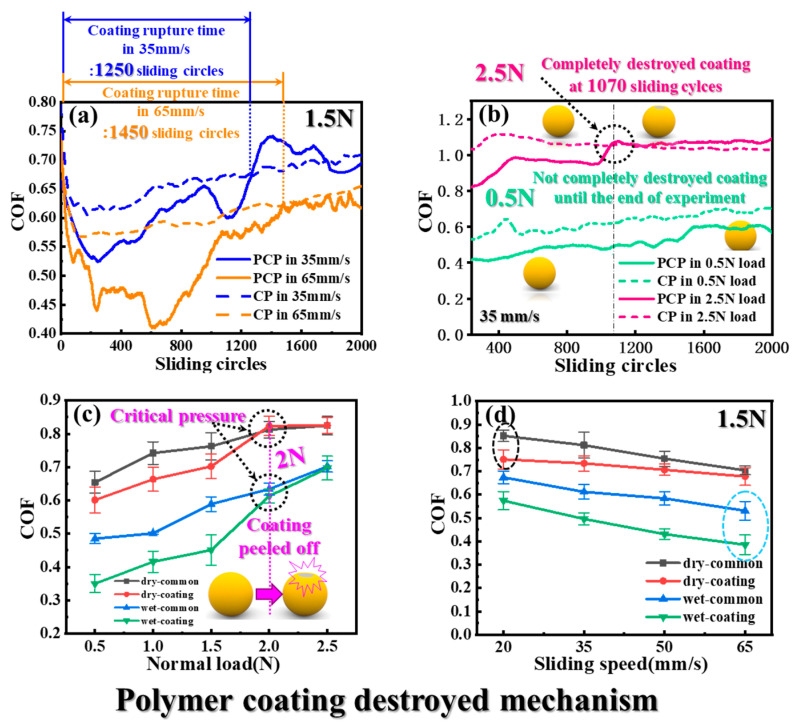
COFs of PCP and CP under different testing loads and speeds: (**a**) COF variations under different speeds at the same loading of 1.5 N. (**b**) COF variations under different loading at the same speed of 35 mm/s. (**c**) The COF of five different loading under 35 mm/s. (**d**) The COF of four different sliding speeds under 1.5 N.

In order to design the PCP to quickly enter between the shale cracks and not easily backflow, it is necessary to reveal the COF value and tribology performance according to the pressure and velocity of the flow [29,30]. As shown in Figure 4a, the higher the sliding speed under constant pressure conditions, the lower the COF. Because of the high velocity of the particles being pumped into the crack, the frictional resistance is small, resulting in a greater movement velocity. According to the obtained COF curve, it can be confirmed that the COF of PCP and shale is smaller than that of CP and shale. This means that the frictional resistance of the PCP entering the cracks is small, so it can easily enter the cracks [31]. When the polymer coating layer is worn and destroyed, the COF value rises sharply, and the behavior of the COF value of PCP shows a similar trend to that of CP. As a result of the analysis, it can be seen that the higher the sliding speed at the same pressure due to the change in frictional resistance, the longer it takes for the polymer coating to completely peel off so that the coating rupture time is proportional to the sliding speed. As shown in Figure 4b, when the sliding speed is constant, the COF is proportional to the pressure. In addition, the higher the normal load, the faster the PCP coating layer peels off, so it can be seen that the load acting on the PCP has a great effect on the polymer film breakage rate [32]. When the polymer film is completely destroyed, the value of PCP’s COF tends to resemble the COF of the CP, which can effectively increase the frictional resistance with the shale to prevent the particles from experiencing flowback. At the same sliding speed of 35 mm/s, the polymer coating was completely destroyed in 1070 sliding cycles under 2.5 N, but the polymer coating remained until the end of the experiment under 0.5 N.

In Figure 4c, at the same speed of 35 mm/s, the COF of the PCP and CP both increase with increasing pressure. In the COF curve, it can be seen that the COF of the coated PCP is lower than that of CP, which shows that PCP can enter and migrate with fluid into the crack more easily than CP due to the low coefficient of friction when initially entering the shale crack. When the normal load is near 2 N, it can be expected that this load is the critical pressure point for breaking the polymer coating film, as the coated particles have almost the same coefficient of friction as the uncoated particles. At this critical point, the particle coating layer is peeled off, and the coefficient of friction with the shale increases, making it difficult for particles that have penetrated into the shale crack to flow back from the crack. Figure 4d shows the effects of different sliding speeds on the COF under the same normal load of 1.5 N. The curve shows that the sliding speed is inversely proportional to the COF, and when the PCP is pumped into the crack at high speed, the COF is lowered, and it can easily and quickly penetrate into the crack, which is advantageous for crack propagation. In addition, the fracturing fluid flows backward according to its own gravity factor after entering the fracture gap during the actual hydraulic fracturing process [33,34,35]. Then, due to the reduced amount of fracturing fluid, dry friction can act between the particles and the shale during fracturing, which accelerates the destruction of the polymer film.

**Figure 5:**
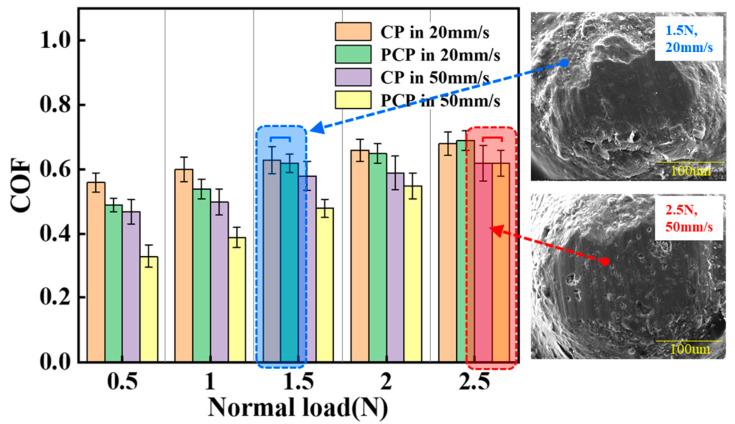
Comparison of friction coefficients between 20 mm/s and 50 mm/s sliding velocities with different load.

In order to better understand the effect of velocity and load on the particle during fracturing, the influence of velocity on proppant under different load conditions was characterized through tribological experimental data. As shown in Figure 5, when the normal load is constant, the friction coefficient of particles is inversely proportional to the sliding speed and the contact angle. Moreover, over a certain load (around 1.5 N), the polymer coating begins to break, which increases the coefficient of friction. A higher sliding speed under the same load results in slightly less damage to the polymer coating. From the blue dotted box, at a sliding speed of 20 mm/s, the COF values of CP and PCP are similar at 1.5 N, which means that the polymer coating is almost peeled off. In the red dotted box, the polymer coating also almost peels off at 2.5 N under 50 mm/s sliding speed. This shows that it is important to reduce the fracturing fluid speed and increase the wall pressure to ensure that more of the coating is removed in the final stages.

### 3.2. Wear Characterization of PCP

**Figure 6:**
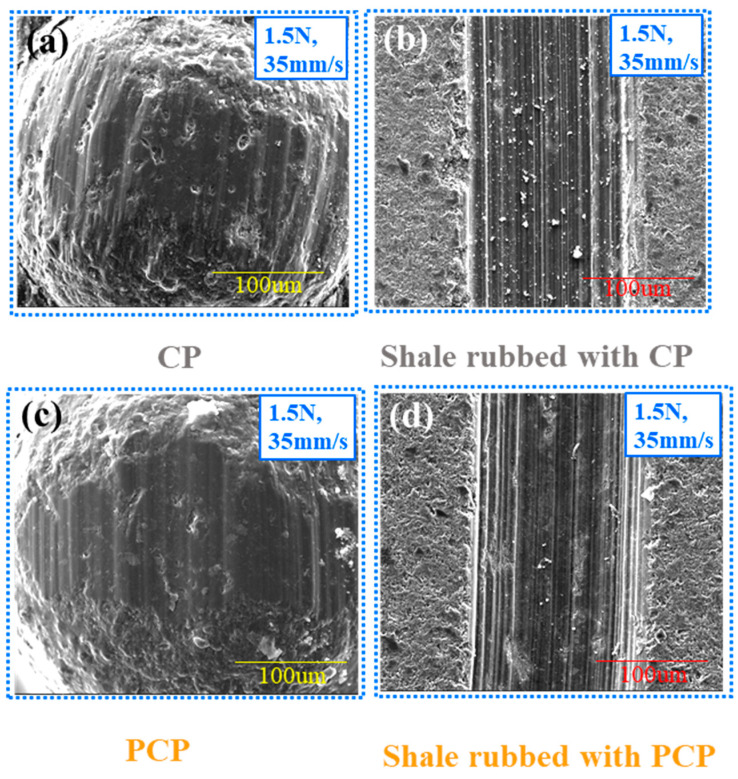
SEM images after fiction and 3D topography in wet condition. (**a**) CP (1.5 N, 35 mm/s). (**b**) Shale rubbed with CP (1.5 N, 35 mm/s). (**c**) PCP (1.5 N, 35 mm/s). (**d**) Shale rubbed with PCP (1.5 N, 35 mm/s).

To comprehensively investigate the effect of velocity and load on particles, the wear profiles of PCP, CP, and shale after friction were compared and analyzed [35]. Compared with the PCP (Figure 6c), the worn surface of the CP (Figure 6a) has more spallation. And the signs of wear on the surface are more obvious on the surface of CP. Since the PCP is coated with a polymer on the surface of the CP, the surface has a lower roughness as well as a lower contact angle, which reduces the degree of friction with the shale. Additionally, the wear type of counterpart is abrasive (Figure 6b,d) and no other types, such as adhesion and furrowing, occur because the conditions are wet. The worn surface of the shale (Figure 6b) has a lot of wear debris, and the wear marks are deeper, indicating that the wear debris has increased the degree of friction with the shale. However, as shown in Figure 6d, the PCP surface has less wear debris, and the wear scars are shallower, indicating that PCP wears slowly in the cracks, which can effectively support the cracks for a long time and reduce the rate of flowback. Thus, polymer coating acted as a lubricant to reduce wear depth. It is more favorable to entry and supports the cracks more than PC.

**Figure 7:**
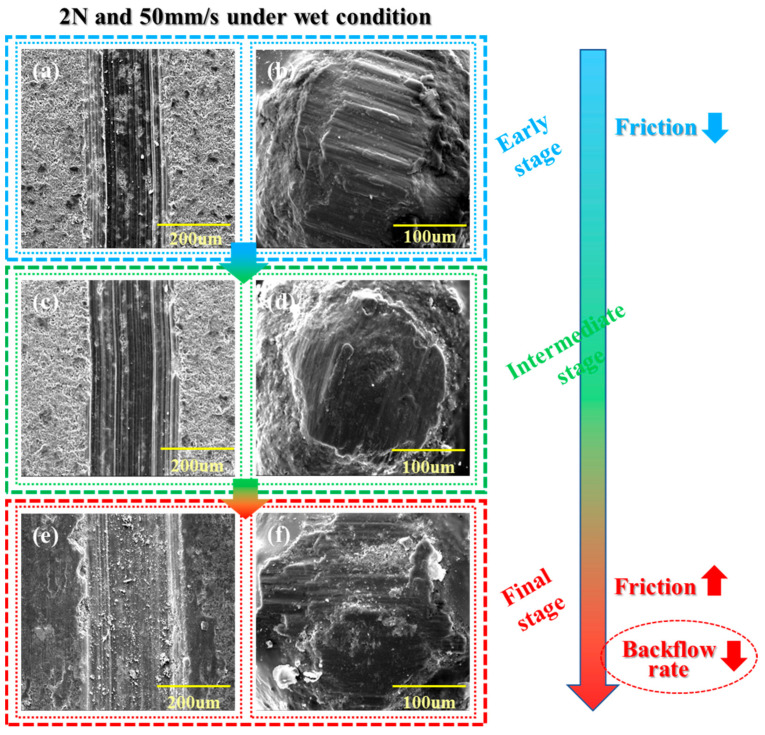
Wear process of PCP and shale under wet friction. (**a**) Shale in the early stage of wear in 2 N and 50 mm/s. (**b**) PCP in the early stage of wear in 2 N and 50 mm/s. (**c**) Shale in the intermediate stage of wear in 2 N and 50 mm/s. (**d**) PCP in the intermediate stage of wear in 2 N and 50 mm/s. (**e**) Shale in the final stage of wear in 2 N and 50 mm/s. (**f**) PCP in the final stage of wear in 2 N and 50 mm/s.

In order to study the wear mechanism of the coating during the fracturing process of PCP, the SEM images of the whole wet friction process were observed and analyzed [36,37]. As shown in Figure 7a, at the early stage of friction, due to the fast-moving speed of PCP, many fragments and slight scratches caused by the friction of polymer film appeared on the surface of the shale. On the PCP surface, fragments from the wear of the polymer film and signs of plastic deformation appeared (Figure 7b). As the friction experiments progressed, it was evident that in the intermediate stage of friction, a large amount of debris was added to the friction medium, resulting in increased friction and increased scratches on the shale surface (Figure 7c). Moreover, some of the ceramsite particles are exposed because of polymer coating damage, increasing the coefficient of friction and facilitating particles to be effectively located deep inside the crack. The shale surface is severely damaged due to repeated abrasion of the polymer film, and much debris appears in the final stage (Figure 7e). Eventually, the polymer film on the contact surface between PCP and the shale was completely worn out and locally disappeared (Figure 7f), resulting in significant leakage scratches, which increased the friction coefficient and prevented particles from experiencing flowback.

**Figure 8:**
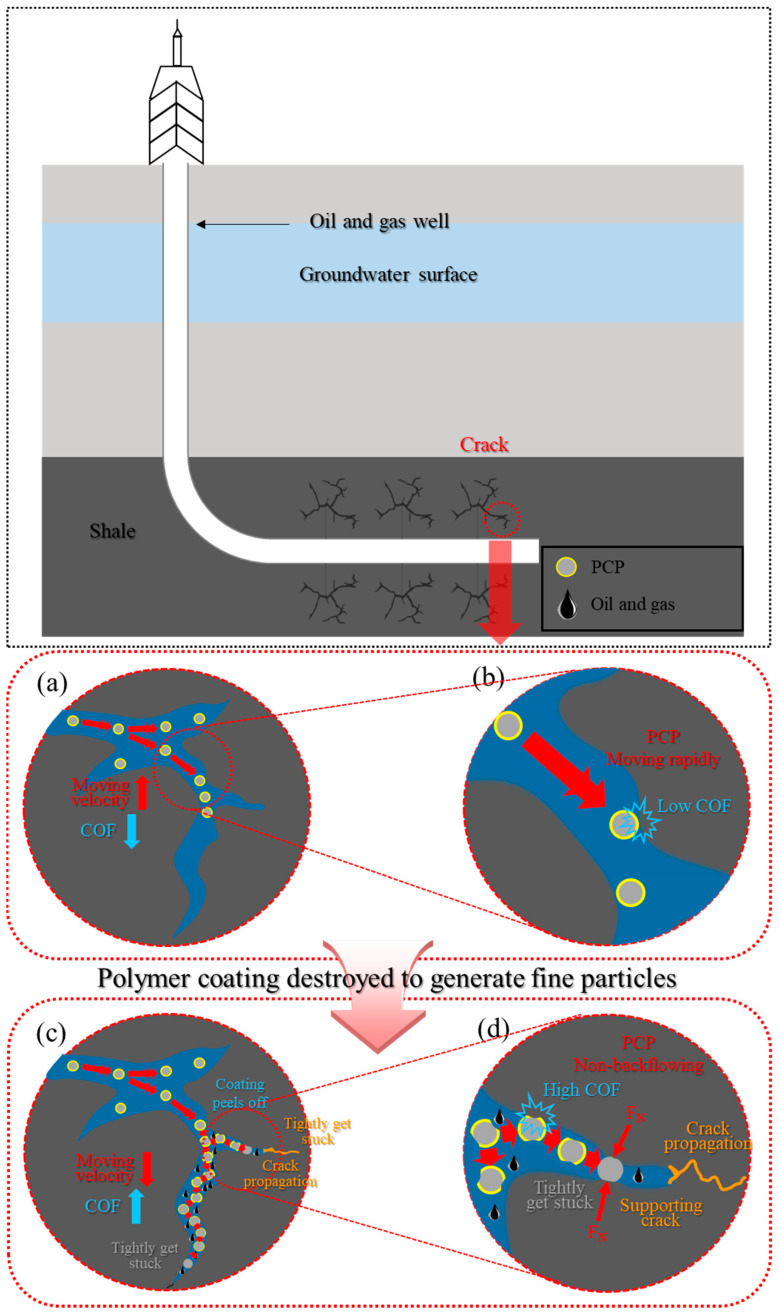
(**a**) The schematic diagram of PCP movement in the crack along with the fracturing fluid (GG). (**b**) The visualization of the rapid movement of PCP in the crack along with the fracturing fluid. (**c**) The schematic diagram of the state of a large number of PCP in the crack. (**d**) The visualization of the PCP under normal load deep inside the crack.

In the actual fracture process, whether particles can effectively support the cracks by being trapped between the cracks without backflow is a decisive factor in the fracturing effect [38,39,40,41,42]. As shown in Figure 8, when a large amount of PCP is pumped into the cracks (Figure 8a), the polymer-coated particles easily enter the cracks together with the fracturing fluid (GG) at high speed due to the high contact angle of the coating (Figure 8b). As PCP enters deep inside the crack, a large number of PCPs move slowly due to reducing fracturing fluid and decreasing COF because the coating layer is destroyed (Figure 8c). As a result, many particles build up deep inside the crack, effectively supporting the crack and allowing the crack to propagate. To recap, the PCP is under pressure from the shale during its movement (Figure 8d). At this pressure, the polymer coating film on the particle surface is destroyed as the PCP comes into contact with the shale for a certain period of time. As the coating film is destroyed, the coefficient of friction with the shale increases, preventing the flowback of the particles supporting the crack and helping further the cracking of the shale rock.

## 4. Conclusions

The comprehensive characterization of the polymer-coated particles was studied through tribological experiments, which showed better hydrophobicity, higher compression resistance, and a lower backflow rate. Material analysis indicated that polymer coatings can be used as lubricants, reducing coefficients of friction and abrasive wear. As a result of the experiment, the sliding distance of polymer coating peeling off is related to the load and the running speed. The sliding distance is 1450 sliding circles at a running speed of 65mm/s and 1070 sliding circles at 2.5N. It was shown that the abrasion of the polymer coating film was small due to the low coefficient of friction when a large amount of polymer coating particles were pumped to the fractured part at high speed. At the same time, the particles can move rapidly with the fracturing fluid in the crack, effectively reducing the time of the particles entering deep inside the crack. In addition, it was confirmed that as the polymer-coated particles entered the crack, the pressure increased, and the coating layer was abraded to increase the COF, thereby successfully preventing the backflow of the particles and supporting the crack. Research into the tribological behavior of PCP will provide strong support for increasing oil extraction yields and improving the industrial environment. Future work can be carried out in terms of both hydrodynamic research and material innovation to provide new ideas for proppant research under hydraulic fracturing.

## Data Availability

Data are contained within the article.

## References

[1] Al-Muntasheri G.A. A critical review of hydraulic fracturing fluids over the last decade. Proceedings of the SPE Western North American and Rocky Mountain Joint Meeting.

[2] Barati R., Liang J.T. (2014). A review of fracturing fluid systems used for hydraulic fracturing of oil and gas wells. J. Appl. Polym. Sci..

[3] Tan P., Jin Y., Han K., Hou B., Chen M., Guo X., Gao J. (2017). Analysis of hydraulic fracture initiation and vertical propagation behavior in laminated shale formation. Fuel.

[4] Yang L., Wang D., Guo Y., Liu S. (2019). Friction and wear behaviors of sand particle against casing steel. Proc. Inst. Mech. Eng. Part J J. Eng. Tribol..

[5] Gallegos T.J. (2018). Hydraulic fracturing (fracking). Access Science.

[6] Boudet H.S., Zanocco C.M., Howe P.D. (2018). The effect of geographic proximity to unconventional oil and gas development on public support for hydraulic fracturing. Risk Anal..

[7] Guo T., Nisbet E.C., Martin J.F. (2019). Identifying mechanisms of environmental decision-making: How ideology and geographic proximity influence public support for managing agricultural runoff to curb harmful algal blooms. J. Environ. Manag..

[8] Li L., Tan J., Wood D.A., Zhao Z., Becker D., Lyu Q., Shu B., Chen H. (2019). A review of the current status of induced seismicity monitoring for hydraulic fracturing in unconventional tight oil and gas reservoirs. Fuel.

[9] Montgomery C.T., Smith M.B. (2010). Hydraulic fracturing: History of an enduring technology. J. Pet. Technol..

[10] Pangilinan K.D., Al C.C., Advincula R.C. (2016). Polymers for proppants used in hydraulic fracturing. J. Pet. Sci. Eng..

[11] Cannan C., Roper T., Savoy S., Mitchell D.R. (2019). Electrically-Conductive Proppant and Methods for Making and Using Same. Google Patents.

[12] Parke M.A., Ramurthy K., Sanchez P.W. New proppant for hydraulic fracturing improves well performance and decreases environmental impact of hydraulic fracturing operations. Proceedings of the SPE Eastern Regional Meeting.

[13] Alary J.A., Parias T. (2013). Method of Manufacturing and Using Rod-Shaped Proppants and Anti-Flowback Additives. Google Patents.

[14] Zhang C., Zhao L., Yu D., Liu G., Pei Y., Huang F., Liu B. (2019). The evaluation on physical property and fracture conductivity of a new self-generating solid proppant. J. Pet. Sci. Eng..

[15] Hussain H., McDaniel R.R., Callanan M.J. (2003). Proppants with Fiber Reinforced Resin Coatings. U.S. Patent.

[16] Burukhin A.A., Kalinin S., Abbott J., Bulova M., Wu Y.K., Crandall M., Kadoma I., Begich M., Papp S. Novel interconnected bonded structure enhances proppant flowback control. Proceedings of the SPE International Symposium and Exhibition on Formation Damage Control.

[17] Monastiriotis S., McCrary A.L., McDaniel R.R., Barthel R.E. (2017). Proppant with Composite Coating. U.S. Patent.

[18] McCrary A.L., McDaniel R.R., Barthel R.E., Monastiriotis S. Proppant with Polyurea-Type Coating. U.S. Patent.

[19] Guo Z., Li X.-Y., Liu C., Feng X., Shen Y. (2013). A shale rock physics model for analysis of brittleness index, mineralogy and porosity in the Barnett Shale. J. Geophys. Eng..

[20] Yang L., Wang D., Guo Y. (2018). Frictional behaviors of iron based tools-casing with sand deposition. Tribol. Int..

[21] Zhang Z., Guo Y., Han F., Wang D., Zhang S. (2021). Multilayer graphene for reducing friction and wear in water-based sand cleaning liquid. Wear.

[22] He H., Luo L., Senetakis K. (2020). Effect of normal load ans shearing velocity on the interface friciton of organic shale-Proppant simulant. Tribol. Int..

[23] Guo Y., Zhang Z., Zhang S.W. (2019). Advances in the application of biomimetic surface engineering in the oil and gas industry. Friction.

[24] Mudgil D., Barak S., Khatkar B.S. (2014). Guar gum: Processing, properties and food applications-a review. J. Food Sci. Technol..

[25] He H., Senetakis K. (2020). A micromechanical study of shale rock-proppant composite interface. J. Pet. Sci. Eng..

[26] He B., Lee J., Patankar N.A. (2004). Contact angle hysteresis on rough hydrophobic surfaces. J. Colloids Surf. A Physicochem. Eng. Asp..

[27] Karim A.M., Rothstein J.P., Kavehpour H.P. (2018). Experimental study of dynamic contact angles on rough hydrophobic surfaces. J. Colloid Interface Sci..

[28] An T., Deng X., Gao Y., Liu S., Dou C., Ju J. (2018). Preparation of highly hydrophobic CeO_2_ films using glancing angle deposition. Mater. Lett..

[29] Yu W., Zhang T., Du S., Sepehrnoori K. (2015). Numerical study of the effect of uneven proppant distribution between multiple fractures on shale gas well performance. Fuel.

[30] Sun J., Schechter D. (2015). Investigating the effect of improved fracture conductivity on production performance of hydraulically fractured wells: Field-case studies and numerical simulations. J. Can. Pet. Technol..

[31] Zheng X., Chen M., Hou B., Ye Z., Wang W., Yin C., Chen X. (2017). Effect of proppant distribution pattern on fracture conductivity and permeability in channel fracturing. J. Pet. Sci. Eng..

[32] Zhong Y., Kuru E., Zhang H., Kuang J., She J. (2019). Effect of fracturing fluid/shale rock interaction on the rock physical and mechanical properties, the proppant embedment depth and the fracture conductivity. Rock Mech. Rock Eng..

[33] Zoveidavianpoor M., Gharibi A. (2015). Application of polymers for coating of proppant in hydraulic fracturing of subterraneous formations: A comprehensive review. J. Nat. Gas Sci. Eng..

[34] Ueno K., Kunisad K., Yamada A.K., Tomita T., Mori K., Iwasa T. (2015). Well Proppant and Method for Recovering Hydrocarbon from Hydrocarbon-Bearing Formation. U.S. Patent.

[35] Green J.W., Terracina J.M., Borges J.F., Spillars S.E., Mah S.H. (2018). Proppant Materials and Methods of Tailoring Proppant Material Surface Wettability. U.S. Patent.

[36] Fu L., Zhang G., Ge J., Liao K., Jiang P., Pei H., Li X. (2016). Surface modified proppants used for porppant flowback control in hydraulic fracturing. Colloids Surf. A Physicochem. Eng. Asp..

[37] Fan F., Feng-Xia L., Shou-Ceng T., Mao S., Waleed K., Ai-Ping S., Yang Z., Quan X. (2021). Hydrophobic epoxy resin coated proppants with ultra-high self-suspension ability and enhanced liquid conductivity. Pet. Sci..

[38] Guo T., Wang Y., Du Z., Chen M., Liu D., Liu X., Rui Z. (2021). Evaluation of coated proppant unconventional performance. Energy Fuels.

[39] Almond S.W., Penny G.S., Conway M.W. Factors affecting proppant flowback with resin coated proppants. Proceedings of the SPE European Formation Damage Conference and Exhibition.

[40] Haque M.H., Saini R.K., Sayed M.A. Proceedings of the Nano-composite resin coated proppant for hydraulic fracturing//Offshore Technology Conference.

[41] Zhang J., Liu K., Cao M. (2017). Experimental study on modified polyacrylamide coated self-suspending proppant. Fuel.

[42] Xie X., Niu S., Miao Y., Gao X., Cheng L., Gao F. (2019). Preparation and properties of resin coated ceramic proppants with ultra light weight and high strength from coal-series kaolin. Appl. Clay Sci..

